# Infectious Mimics of Bell's Palsy: Facial Nerve Palsy Due to Lyme Neuroborreliosis

**DOI:** 10.7759/cureus.93122

**Published:** 2025-09-24

**Authors:** Tha Mon, Aye Nyein, Aung Oo

**Affiliations:** 1 Internal Medicine, University Hospital Birmingham, Birmingham, GBR; 2 Internal Medicine, Blackpool Teaching Hospitals, Blackpool, GBR

**Keywords:** bilateral facial palsy, facial nerve palsy, infectious etiology, lyme neuroborreliosis, lyme serology

## Abstract

Facial nerve palsy (FNP) is a common neurological disorder. There are multiple causes of FNP, and Bell’s palsy is defined as an idiopathic cause of FNP. If a patient presents with facial palsy, a full investigation workup should be performed. Lyme disease can present with erythema migrans, fever, headache, muscle and joint pain, and facial palsy. We present a case of a 47-year-old man who presented with progression from unilateral to bilateral FNP. After a series of investigations, he was diagnosed with LNB. Even if MRI findings are normal, cerebrospinal fluid (CSF) analysis and Lyme serology (enzyme-linked immunosorbent assay followed by Western blot) in serum and/or CSF can confirm the diagnosis of neuroborreliosis. We have administered the patient IV ceftriaxone 2 g daily, as per the guidelines. He showed gradual improvement in FNP from House-Brackmann Grade V to Grade II on subsequent follow-ups. This case emphasizes the importance of broad clinical evaluation in diagnosing FNP. Early detection of LNB and initiation of treatment can ensure patients have a favorable outcome.

## Introduction

Facial nerve palsy (FNP) is classified as either peripheral (lower motor neuron) or central (upper motor neuron). Because both sides of the cerebral hemispheres provide signals to the forehead muscles, central lesions that impair the corticobulbar pathways usually spare the forehead. However, the peripheral lesions of cranial nerve VII affect both the upper and lower facial muscles on one side. It causes classic symptoms such as facial drooping, loss of forehead wrinkling, difficulty closing the eyes, slurred speech, and impaired emotional expression [1].

Idiopathic Bell’s palsy is the most common cause of peripheral FNP, accounting for 60-70% in some studies [1,2]. Most patients achieve recovery spontaneously, and starting corticosteroid medication within 72 hours significantly improves outcomes [2]. Bell’s palsy is a diagnosis of exclusion, and broad differential diagnoses should be considered, especially when patients present with unusual symptoms such as bilateral facial palsy or systemic symptoms, including skin rash, joint pains, sensory impairment, or motor weakness in the body. The differential diagnoses include Lyme neuroborreliosis (LNB), human immunodeficiency virus (HIV) infection, Ramsay Hunt syndrome, otitis media, sarcoidosis, neoplasms, or stroke [2,3].

In regions where LNB is endemic, the most common neurological sign is involvement of cranial nerve VII on one side or both sides [4,5]. As LNB is a clinical mimic of idiopathic Bell’s palsy, it can be overlooked unless appropriate investigations such as enzyme-linked immunosorbent assay (ELISA), confirmatory Western blot, and cerebrospinal fluid (CSF) analysis are performed. Additionally, LNB should be treated early with IV ceftriaxone or oral doxycycline for 14-21 days [5]. Our case highlights the importance of awareness of infectious etiologies, particularly LNB, to achieve early diagnosis and targeted treatment to prevent long-term neurologic sequelae.

## Case presentation

Demographics and medical history

A 47-year-old man presented with right-sided facial palsy two weeks after returning from Greece. He was initially treated for Bell’s palsy with corticosteroids. Ten days later, he re-presented with bilateral facial palsy, which had developed in the preceding five days. He noted that his mouth was drooping on both sides, and his eyes were unable to close as he wished. He had no history of headache, dizziness, hearing difficulty, slurred speech, swallowing difficulties, limb weakness, numbness, or tingling. He denied experiencing infectious symptoms, such as fever, cough, diarrhea, and sore throat. He did not recall visiting rural areas or experiencing insect bites during his time in Greece. Apart from well-controlled hypertension, no other past medical history was found.

The examination showed no rash or edema of the joints. During a neurological assessment, it was found that the patient had bilateral lower motor neuron facial palsy. He could not frown, blow up his cheeks, or clench his teeth. The corneal reflexes were normal; however, the eyelids on both sides were not fully closed. The other cranial nerves were intact. There were no symptoms of cerebellar lesions, the auditory canals and tympanic membranes were unremarkable, and there were no specific motor or sensory deficits in the limbs.

Investigations

At the time of the first presentation with right-sided Bell’s palsy, the patient’s routine blood tests, including liver function, thyroid function, renal function, and full blood count, were all within normal limits (Table 1).

**Table 1:** Normal blood test results CRP: C-reactive protein, eGFR: estimated glomerular filtration rate, TSH: thyroid-stimulating hormone, HbA1C: glycated haemoglobin

Blood tests	Result	Normal range
Hemoglobin	147	130-180 g/L
White cell count	5.97	4.0-11.0 x 10^9^/L
Platelets	291	150-400 x 10^9^/L
Neutrophils	2.7	1.8-7.7 x 10^9^/L
Lymphocytes	2.63	1.4-4.8 x 10^9^/L
Monocytes	0.38	0.1-0.8 x 10^9^/L
Eosinophils	0.24	0.1-0.6 x 10^9^/L
Basophils	0.1	0-0.1 x 10^9^/L
CRP	<1	<5 mg/L
Sodium	143	133-146 mmol/L
Potassium	4	3.5-5.3 mmol/L
Urea	3.6	2.5-7.8 mmol/L
Creatinine	83	59-104 mmol/L
eGFR	>90	>60 ml/min
Albumin	37	35-50 g/L
Total protein	64	60-80 g/L
Bilirubin	4	0-21 umol/L
Alanine transaminase	40	<41 IU/L
Alkaline phosphatase	65	30-130 IU/L
TSH	1.3	0.4-4.0 mIU/L
HbA1C	37	48-58 mmol/mol

**Figure 1:**
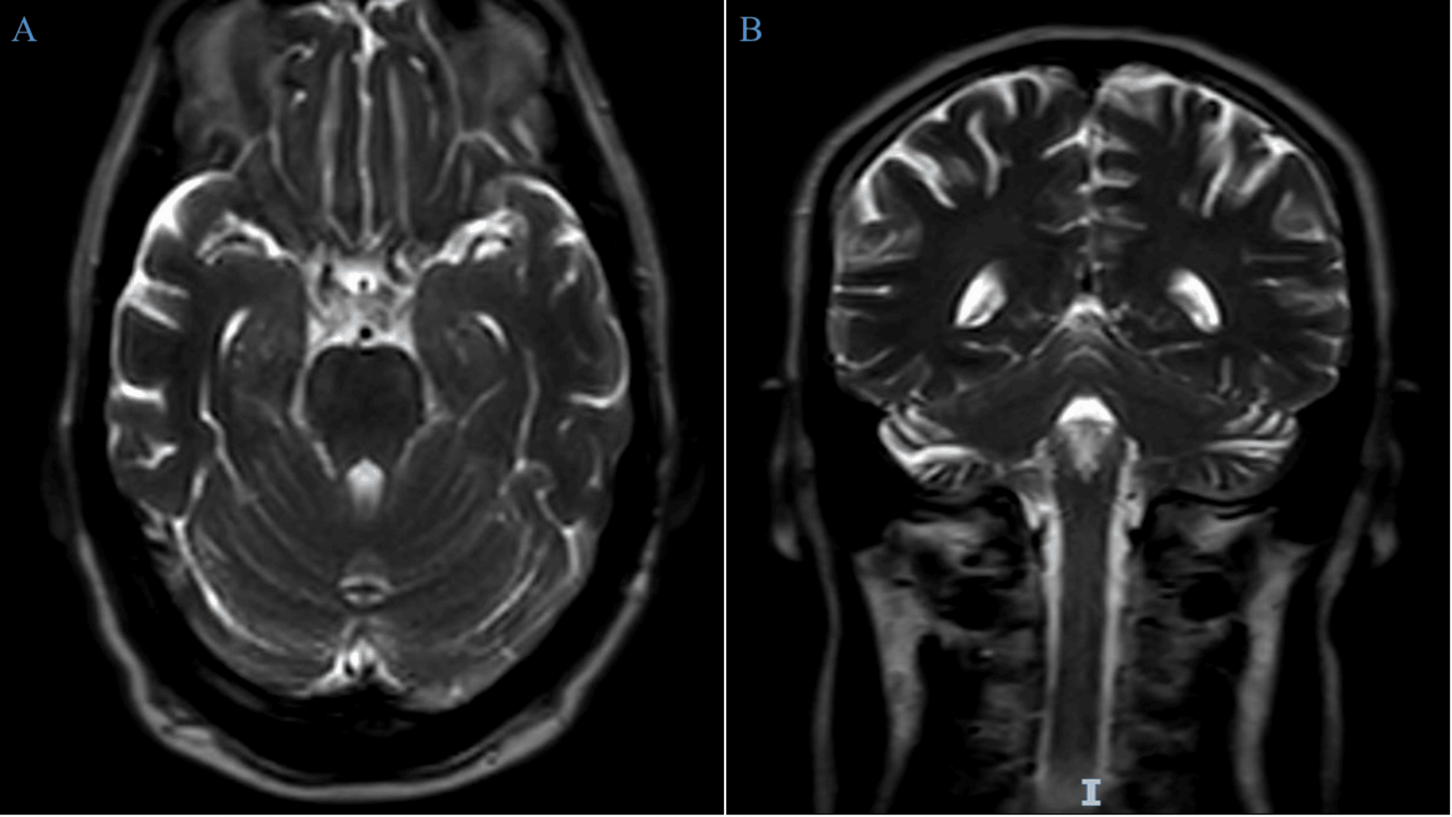
(A, B) MRI of the head with contrast MRI: magnetic resonance imaging

On the second presentation with bilateral facial palsy, we performed systematic investigations. MRI of the brain showed no abnormal enhancement or lesions after IV contrast administration (Figure 1).

**Figure 2:**
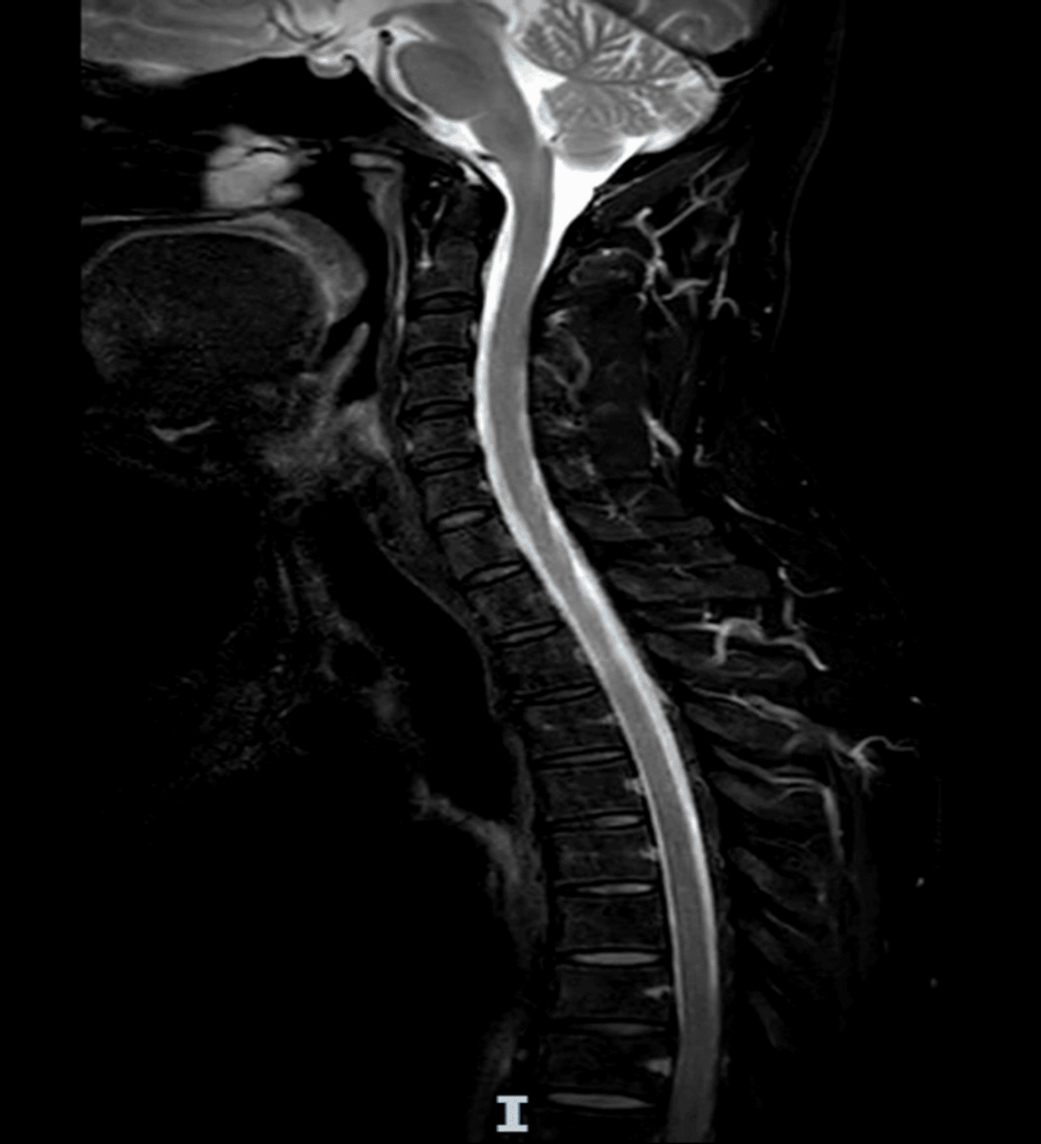
MRI of the whole spine MRI: magnetic resonance imaging

An MRI of the whole spine was conducted to rule out myelitis, which revealed no evidence of myelitis or other significant abnormality to explain the patient's symptoms (Figure 2).

Subsequently, an infection screening, including HIV, hepatitis B, hepatitis C, herpes simplex virus, varicella zoster virus, and enterovirus, as well as an autoimmune panel that included antinuclear antibody (ANA), antineutrophil cytoplasmic antibody, and anti-double-stranded DNA antibody, all returned negative results.

Lyme serology was performed when the patient re-presented with bilateral FNP, 10 days after the initial presentation. A two-tiered approach was used, with initial ELISA testing followed by confirmatory Western blot after one week, which was positive. We performed a lumbar puncture to rule out neurological infections. CSF analysis revealed lymphocytic pleocytosis, elevated protein levels, and normal glucose. CSF immunoblot revealed intrathecal production of Borrelia-specific antibodies.

The patient's history of travelling to an area where Lyme disease is endemic, having bilateral palsy, and positive blood and CSF serology findings led to a definitive diagnosis of neuroborreliosis. He was admitted and treated with a 21-day course of IV ceftriaxone (2 g once a day). To avoid exposure keratitis, the supportive treatment included eye lubrication and protective eye patches at night. He was under the joint care of the neurology and infectious diseases teams. He recovered gradually from severe facial palsy to mild residual weakness by six months of follow-up.

## Discussion

Bell’s palsy not only affects physical health but also can have an impact on mental health. It is crucial to maintain clinical vigilance in this regard. If a patient presents with unusual symptoms along with a systemic insult, it is essential to conduct comprehensive investigations to identify the potential causes of facial palsy (Table 2) [6,7].

**Table 2:** Causes of facial palsy [8]

Category	Notable factors
Infectious	*Borrelia burgdorferi*, herpes simplex virus, varicella-zoster virus, Epstein-Barr virus, HIV, influenza, mumps, *Mycobacterium tuberculosis*, *Mycobacterium leprae*, *Corynebacterium diphtheriae*, *Clostridium tetani*, malaria, *Treponema pallidum*
Ear infections	Acute suppurative otitis media, cholesteatoma
Metabolic	Diabetes, hypertension, dyslipidaemia, obesity, acute porphyria, alcoholic neuropathy
Autoimmune	Systemic lupus erythematosus, sarcoidosis, amyloidosis, Behcet’s disease, periarteritis nodosa
Hormonal	Pregnancy
Environmental triggers	Cold exposure, seasonal change, stress
Neurological	Multiple sclerosis, Guillain-Barré syndrome, benign intracranial hypertension, brain tumor, myasthenia gravis
Congenital	Myotonic dystrophy, Moebius syndrome, facial musculature absence

As Bell's palsy is a diagnosis of exclusion, a detailed medical history that includes risk factors, travel history, sexual exposure, and systemic symptoms must be explored. It is essential to consider the differential diagnosis of bilateral FNP. Neuroimaging (MRI with contrast) is needed to rule out central causes such as tumors or strokes, or nerve compression. If there is a suspicion of a central or infectious cause, such as Lyme disease or neurosyphilis, a lumbar puncture is indicated [9]. Early initiation of targeted treatment is critical: IV ceftriaxone for neuroborreliosis, antiretroviral therapy for HIV, and IV immunoglobulin or plasma exchange for GBS. In addition, eye tape or artificial tears should be used to help protect the cornea. Engaging the patient in physiotherapy can help recover functionally [8,10].

Lyme disease can affect the nervous system in both its early, localized stages and later, generalized stages. Facial palsy occurs in 11% of neuroborreliosis cases, with 30-40% of those being bilateral [10]. The diagnosis is primarily based on clinical evidence but is also supported by serological tests, which involve a two-tier approach: ELISA followed by Western blot, demonstrating sensitivity and specificity for IgM and IgG [11]. The Infectious Disease Society of America recommended IV ceftriaxone or oral doxycycline for 14 to 21 days. The choice of antibiotics depends on the patient's allergy status and tolerance to oral medications [5]. According to Kortela et al., oral doxycycline is just as effective as IV ceftriaxone for treating neuroborreliosis [12]. The role of corticosteroids remains controversial, as per Avellan and Bremell [13]. Early initiation of antibiotics can achieve recovery in up to 72% of cases within a few months [14]. Post-treatment Lyme disease syndrome, which includes residual cranial neuropathy, fatigue, and cognitive impairments, can happen in 10-20% of patients [15].

Our case was diagnostically challenging because the patient did not exhibit the hallmarks of Lyme disease, such as erythema migrans, myalgia, arthralgia, fever, or headache, and he did not recall any tick bite while travelling in Greece. He was initially started on corticosteroids for right-sided facial palsy, assuming Bell’s palsy. However, he developed bilateral facial palsy, which is atypical of Bell’s palsy. This leads to thorough investigations, including neuroimaging, CSF analysis, infection screening, and serology, which confirmed the diagnosis of LNB. He was promptly started on ceftriaxone, and no additional corticosteroid course was given. He recovered gradually from facial palsy on follow-up at a later stage. This case highlights an unusual presentation of LNB and underscores the importance of maintaining a high clinical suspicion in diagnosing Bell’s palsy.

There are two comparable case reports highlighting the diagnostic dilemma of LNB in the absence of typical clinical features. One case described the event of neuroborreliosis without dermatological manifestations [16], whilst the other case reported Borrelia infection presenting with stroke without other cranial neuropathy [17], both showcasing the diverse and atypical presentation of Lyme disease. Our case enriched this spectrum with the event of progression from unilateral to bilateral FNP in the absence of systemic symptoms or tick exposure. Nevertheless, the intrinsic limitations of a single case report must be acknowledged: patient recall of tick exposure can be unreliable, the findings may not be widely generalizable, and the absence of certain ancillary investigations may limit diagnostic completeness.

## Conclusions

LNB presenting with progressive FNP could be diagnostically complex, especially in the absence of typical clinical features of Lyme disease. Clinicians should maintain a high index of clinical suspicion and consider a wide range of differential diagnoses when treating FNP. Neuroborreliosis should be taken into account in the initial investigation of unilateral or bilateral FNP regardless of tick exposure, which has limited reliability. Early recognition helps in the prompt initiation of treatment and may prevent chronic neurological deficits. Involving a multidisciplinary team, including neurologists, physicians, infectious disease specialists, and physiotherapists, plays a vital role in optimizing patient care.
